# Chaotic dynamics in spatially distributed neuronal networks generate population-wide shared variability

**DOI:** 10.1371/journal.pcbi.1010843

**Published:** 2023-01-10

**Authors:** Noga Mosheiff, Bard Ermentrout, Chengcheng Huang

**Affiliations:** 1 Department of Neuroscience, University of Pittsburgh, Pittsburgh, Pennsylvania, United States of America; 2 Center for the Neural Basis of Cognition, Pittsburgh, Pennsylvania, United States of America; 3 Department of Mathematics, University of Pittsburgh, Pittsburgh, Pennsylvania, United States of America; Inria, FRANCE

## Abstract

Neural activity in the cortex is highly variable in response to repeated stimuli. Population recordings across the cortex demonstrate that the variability of neuronal responses is shared among large groups of neurons and concentrates in a low dimensional space. However, the source of the population-wide shared variability is unknown. In this work, we analyzed the dynamical regimes of spatially distributed networks of excitatory and inhibitory neurons. We found chaotic spatiotemporal dynamics in networks with similar excitatory and inhibitory projection widths, an anatomical feature of the cortex. The chaotic solutions contain broadband frequency power in rate variability and have distance-dependent and low-dimensional correlations, in agreement with experimental findings. In addition, rate chaos can be induced by globally correlated noisy inputs. These results suggest that spatiotemporal chaos in cortical networks can explain the shared variability observed in neuronal population responses.

## Author summary

Cortical neurons exhibit highly variable spatiotemporal activity patterns, even in the presence of strong sensory stimuli. The patterns of population responses also vary depending on animal’s brain state and behavioral context. Understanding the dynamical properties of cortical circuit is critical to understanding network’s responses to stimulus inputs. Previous models have proposed that neural variability can be generated through chaotic neural dynamics in cortical circuits. However, these models fail to capture the statistical features of the shared variability in population activity patterns observed in experiments. In this work, we systematically analyze the dynamical regimes of spatially distributed neuronal networks. We discover spatiotemporal chaos that produces correlated response patterns in neural population activity, consistent with cortical recordings. Interestingly, we find chaos in networks where the excitatory and inhibitory neurons have similar spatial spreads of connections, which is an anatomical feature of sensory cortex. We further show that chaotic activity can be induced when the network is driven by correlated noisy inputs, which can explain the prevalence of large-scale shared variability observed in cortex. Our work expands our mechanistic understanding of neural dynamics in cortical circuit models.

## Introduction

A defining feature of cortical neural responses is that they are highly variable. The variability is reflected at multiple scales of neural recordings, from irregular inter-spike intervals in spike trains of individual neurons [1, 2] to spatiotemporal patterns in mesoscopic neural activity measured with voltage sensitive dye imaging [3] and local field potentials [4], to whole brain signals such as the electroencephalography [5]. Changes in neural variability reflect fluctuations in the brain state and are closely related to behavioral performance [5–8]. Therefore, understanding the circuit mechanisms that generate neural variability is critical for elucidating the neural basis of behavior.

Previous models have proposed that chaotic neural dynamics can be a substantial local source of neural variability in cortical circuits [9–11]. Variable neural responses can be intrinsically generated through strong interactions between the excitatory and inhibitory neurons. Intriguingly, neuronal networks with chaotic dynamics have been shown to demonstrate high computational capabilities because of their rich reservoir of internal dynamics that can be utilized for complex computations and efficient training [12–16].

However, previous models with unstructured random connectivity produce chaotic response in individual neurons that is uncorrelated with other neurons in the network. In contrast, numerous datasets of cortical recordings have revealed that cortical neurons are on average positively correlated [17]. The correlation between a pair of neurons depends on many factors, such as the cortical distance between them and their tuning similarity [18–20]. Moreover, the variability shared among a neuron population has been found to be low dimensional, meaning that the variations in population response patterns can often be explained by just a few independent latent variables [21–25]. Therefore, networks with unstructured random connectivity are not able to capture the shared variability in neural population responses.

A main determinant of the connection probability between a pair of neurons in cortex is the physical distance between them [26–30]. Nearby neurons are more likely to be connected, whereas neurons that are far apart are less likely to be connected. Recently, several studies of spatially distributed neuronal networks have suggested that spatiotemporal patterns of neural activity can explain many features of variability in neural population responses [21, 31–33]. For example, our past work has shown that spiking neuron networks with irregular wave dynamics generate on average positive correlations and low-dimensional population-wide shared variability, consistent with cortical recordings [21].

Here we systematically analyze the dynamical regimes of spatially distributed firing rate networks. We find a parameter region where networks exhibit irregular chaotic dynamics. The chaotic solutions have several features of response variability that are consistent with experimental findings in cortex, such as broadband frequency power in single neuron responses, distance-dependent correlations and low-dimensionality of population responses. Interestingly, chaos occurs in networks where the excitatory and inhibitory neurons have similar projection widths, an anatomical feature found in the cortex [27, 29, 30]. Further, we find that correlated noisy inputs induce chaos, which can explain the prevalence of large-scale shared variability observed in cortex. Our work identifies a new dynamical regime of spatiotemporal chaos in neuronal networks that can account for rich response patterns in neural population activity.

## Results

**Fig 1:**
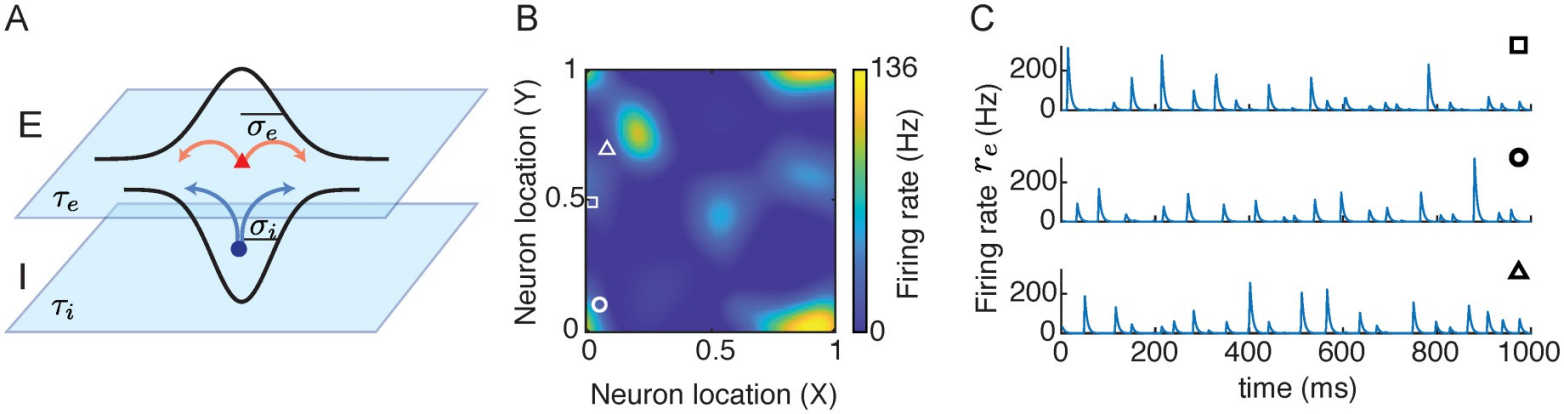
Irregular spatiotemporal dynamics in spatially distributed networks. **A**. Model schematic of recurrently coupled excitatory (E) and inhibitory (I) neurons. Neurons from each population are equally spaced on a two dimensional sheet, [0, 1] × [0, 1], with distance-dependent connectivity weights. **B**. A snapshot of the firing rates of excitatory neurons. **C**. Three examples of the time courses of firing rates of neurons at different locations. The square, circle and triangle in panel **B** denote the spatial location of each neuron.

We study a spatially distributed network model that describes the firing rate dynamics of the excitatory (*r*_*e*_) and inhibitory (*r*_*i*_) neurons (Eqs 1 and 2). Neurons are organized on a two-dimensional sheet (*x* ∈ [0, 1] × [0, 1]) with periodic boundary conditions (Fig 1A). The equations that describe the dynamics of the firing rates are

$$\begin{array}{ccc} \tau_{e}\frac{\partial r_{e}\left(x,t\right)}{\partial t} & = & -r_{e}+\phi (W_{ee}g\left(x,\sigma_{e}\right)*r_{e}+W_{ei}g\left(x,\sigma_{i}\right)*r_{i}+\mu_{e}), \end{array}$$ (1)

$$\begin{array}{ccc} \tau_{i}\frac{\partial r_{i}\left(x,t\right)}{\partial t} & = & -r_{i}+\phi (W_{ie}g\left(x,\sigma_{e}\right)*r_{e}+W_{ii}g\left(x,\sigma_{i}\right)*r_{i}+\mu_{i}), \end{array}$$ (2)

where *τ*_*e*_ (*τ*_*i*_) is the time constant of the excitatory (inhibitory) population, * denotes convolution in space, and *ϕ*(*x*) = max(*x*, 0)^2^ is the input-output transfer function of each neuron. The connection strength from a neuron in population *β* to a neuron in population *α* decays with distance as a Gaussian function, *g*(*x*, *σ*_*β*_), with projection width *σ*_*β*_ (*α*, *β* = *e*, *i*). The average connection strength from population *β* to population *α* is *W*_*αβ*_. The external input to each population is a static and spatially homogeneous current, *μ*_*α*_ (*α* = *e*, *i*).

The spatial networks generate rich spatiotemporal patterns. In particular, we find that the network exhibits irregular patterns in both space and time for certain parameters (Fig 1B and 1C). These networks show spatially localized and transient activity patterns that sometimes propagate across the network (Fig 1B). Individual neurons show epochs of brief firing with varying magnitudes and time intervals in between (Fig 1C). This type of network dynamics result in large variability that is shared among neurons in the network. In order to better understand the behavior of the model and the mechanism for generating irregular firing patterns, we systematically analyze the different dynamical regimes of the spatially distributed networks. We focus our analysis on varying the temporal (*τ*_*i*_) and spatial (*σ*_*i*_) scales of the inhibitory neurons, while fixing those of the excitatory neurons (*τ*_*e*_ = 5 ms and *σ*_*e*_ = 0.1).

### A reduced two-unit model with no spatial coupling

We first consider networks with no spatial coupling, meaning that the spatial coupling function, *g*, is constant over distance. In this case, the network is reduced to a two-unit system, where the firing rate, *r*_*α*_(*x*, *t*) = *r*_*α*_(*t*), *α* = *e*, *i*, is independent of the neural location *x*.

$$\begin{array}{ccc} \tau_{e}\frac{dr_{e}}{\text{d}t} & = & -r_{e}+\phi (W_{ee}r_{e}+W_{ei}r_{i}+\mu_{e}), \end{array}$$ (3)

$$\begin{array}{ccc} \tau_{i}\frac{\text{d}r_{i}}{\text{d}t} & = & -r_{i}+\phi (W_{ie}r_{e}+W_{ii}r_{i}+\mu_{i}). \end{array}$$ (4)

Note that a solution to the two-unit system corresponds to a spatially uniform solution to the spatially distributed networks (Eqs 1 and 2).

**Fig 2:**
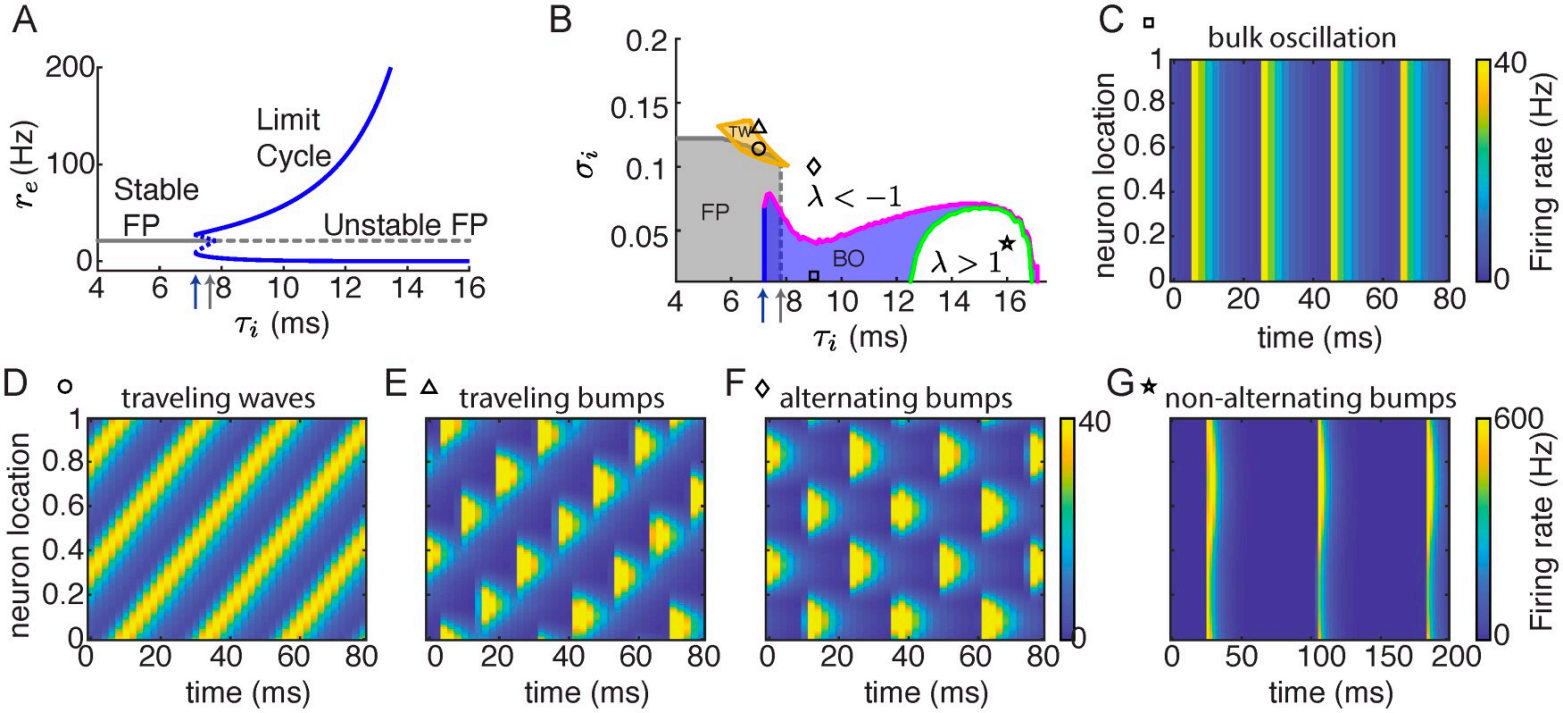
Dynamical regimes of networks with one-dimensional spatial coupling. **A**. Bifurcation diagram of the reduced two-unit model (Eqs 3 and 4) as *τ*_*i*_ varies. Gray line, fixed point; blue curves, the maximal and minimal firing rates of a limit cycle; solid, stable; dashed, unstable. The blue arrow indicates the lowest *τ*_*i*_ with a limit cycle solution. The gray arrow indicates the Hopf bifurcation (the largest *τ*_*i*_ with a stable fixed point solution). **B**. Phase diagram of networks with one dimensional spatial coupling. Gray, stable fixed point; blue, stable bulk oscillation; orange, stable traveling waves. The fixed point solution loses stability at either the zero wave number (gray dashed line) or a nonzero wave number (gray solid line). The bulk oscillation loses stability with either an eigenvalue becoming larger than 1 (green curve) or an eigenvalue becoming less than -1 (magenta curve). Blue and gray arrows point to the same values of *τ*_*i*_ as in **A**. **C-G** Examples of the firing rate dynamics of the excitatory neurons in networks with different *τ*_*i*_ and *σ*_*i*_ (the square, circle, triangle, diamond and star symbols indicate the parameters in panel **B**). **C**, Bulk oscillation; **D**, traveling waves; **E**, traveling bumps; **F**, alternating bumps; **G**, non-alternating bumps. The temporal and spatial scales of the excitatory neurons are *τ*_*e*_ = 5 ms and *σ*_*e*_ = 0.1, respectively.

The reduced network (Eqs 3 and 4) has a stable fixed point solution for a small *τ*_*i*_ (Fig 2A, gray solid line). As *τ*_*i*_ increases, the fixed point solution becomes unstable through a Hopf bifurcation (*τ*_*i*_ = *τ*_HB_, Fig 2A, gray arrow), and the system admits a stable periodic solution (limit cycle; Fig 2A, blue solid curve). Over the interval of *τ*_*i*_ ∈ [7.16, 7.78] ms both the fixed point and the limit cycle solutions are stable (between the blue and gray arrows in Fig 2A). We next analyze the stability of the fixed point and the limit cycle solutions in the spatially distributed networks (Eqs 1 and 2).

### Pattern formation in one-dimensional networks

In this section we analyze the stability of spatially uniform solutions and how a loss of stability leads to periodic spatiotemporal patterns. We first consider networks with one-dimensional spatial coupling, where neurons are distributed over a line interval, [0, 1], with periodic boundary conditions (namely, a ring). The stability analysis below is also applicable to two-dimensional networks.

We first analyze the stability of the fixed point solution in spatially distributed networks, which is a static and spatially uniform solution. We linearize around the fixed point in the spatial frequency domain using Fourier transform and obtain eigenvalues for each wave number (spatial frequency) (see Methods; [34, 35]). The fixed point solution is stable when all eigenvalues are negative (stable region is shown in gray in Fig 2B). When *σ*_*i*_ < *σ*_*e*_, the network loses stability at zero wave number as *τ*_*i*_ increases, which is the same condition as the Hopf bifurcation in the two-unit model (Fig 2B, gray arrow, gray dashed line). For small *σ*_*i*_ and *τ*_*i*_ > *τ*_HB_, the network exhibits spatially uniform and temporally periodic solutions (bulk oscillation; Fig 2C), which corresponds to the limit cycle solution in the two-unit model (Eqs 3 and 4). When *σ*_*i*_ > *σ*_*e*_, the network loses stability at a nonzero wave number (Fig 2B, gray solid line), suggesting pattern formation of spatially and/or temporally periodic solutions.

Around the boundary where the fixed point solution loses stability at a nonzero wave number, we find traveling wave solutions (Fig 2B orange region, Fig 2D). Using a continuation numerical method [36], we show that stable traveling wave solutions exist in a small parameter region (Fig 2B, orange) that partially overlaps with the region of stable fixed point. Closely beyond this region with a larger *σ*_*i*_, the traveling waves lose stability and the networks generate traveling bump solutions (Fig 2E).

We next compute the stability of the bulk oscillation solution. Similar to the stability analysis of the fixed point solution, we linearize around the bulk oscillation solution and perturb the system at different wave number (see Methods). The dynamics of the perturbation then follow a linear system of differential equations with periodic coefficients (Eq 8). The stability of the bulk oscillation solution depends on the eigenvalues (λ) of the monodromy matrix, *M*, of the linear system, which describes the change of the perturbation after one period of the bulk oscillation solution (Eq 9; [37, 38]). The bulk solution is unstable if there is an eigenvalue of *M*(*k*) with magnitude larger than 1 for any wave number *k*. We find that with small *σ*_*i*_ and an intermediate range of *τ*_*i*_, the bulk oscillation is stable (Fig 2B, blue region; Fig 2C). As *σ*_*i*_ increases, the bulk oscillation loses stability with a real eigenvalue less than -1 for perturbations at a nonzero wave number, indicating a period-doubling bifurcation (Fig 2B, magenta curve). In the parameter region beyond this stability boundary, the network activity shows spatial patterns that alternate over time (Fig 2F). As *τ*_*i*_ increases, the bulk oscillation loses stability with a real eigenvalue larger than 1 (Fig 2B, green curve). In the region under this stability boundary (Fig 2B, green curve), the network activity exhibits spatial patterns that repeats at each cycle (Fig 2G).

### Chaotic dynamics in two dimensional networks

**Fig 3:**
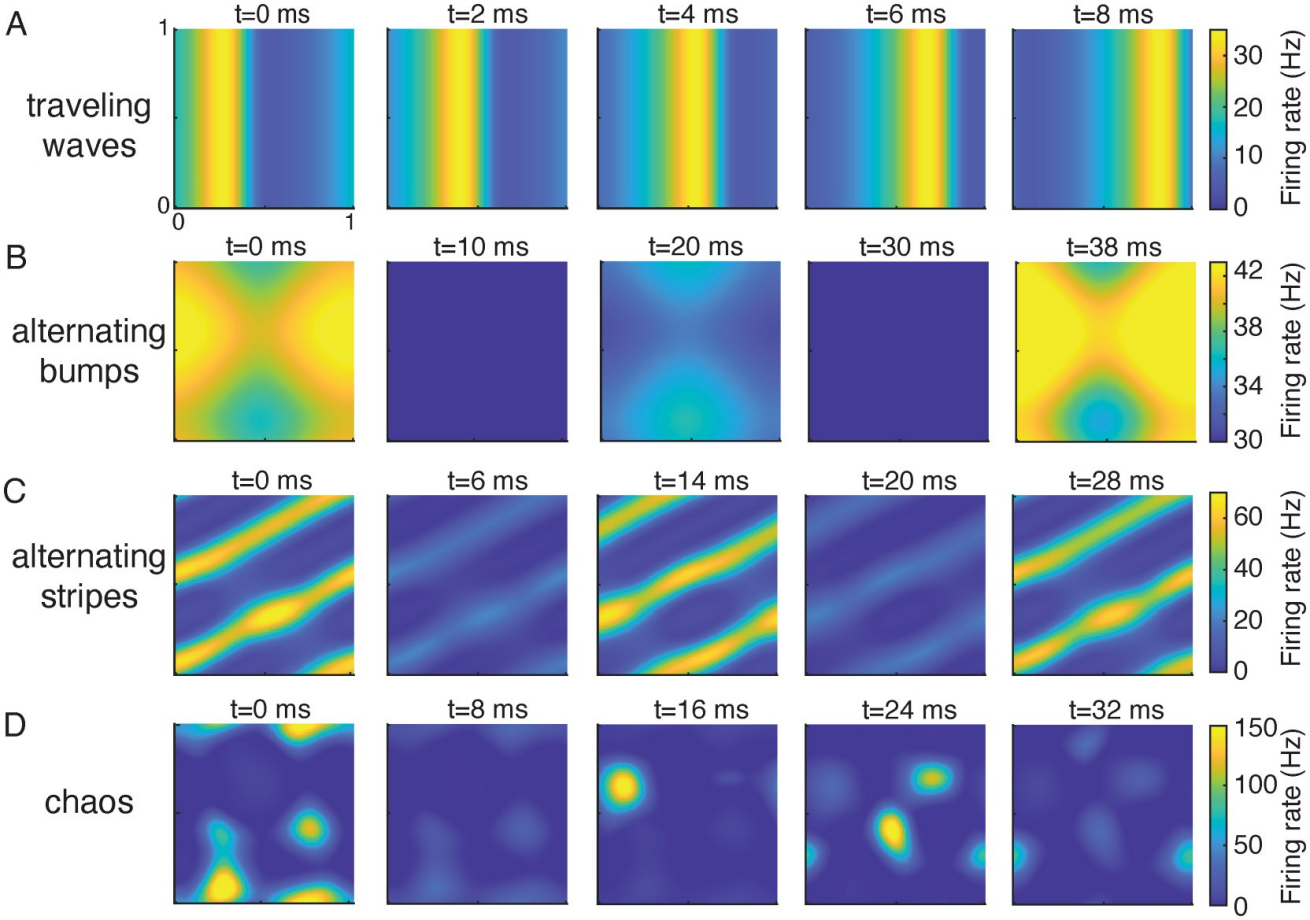
Examples of solutions in networks with two-dimensional spatial coupling. **A-D**, Snapshots of the firing rates of the excitatory population at five time frames. **A**, traveling waves solution (*τ*_*i*_ = 8, *σ*_*i*_ = 0.1). **B**, alternating bumps solution (*τ*_*i*_ = 9, *σ*_*i*_ = 0.06). **C**, alternating stripes solution (*τ*_*i*_ = 9, *σ*_*i*_ = 0.1). **D**, chaotic solution (*τ*_*i*_ = 12.8, *σ*_*i*_ = 0.096). The axes are the neuron location on the two-dimensional neuronal sheet.

We next analyze the full networks with two dimensional spatial coupling (Fig 1A). The stability analysis of the fixed point and the bulk oscillation that we outlined above for the one-dimensional networks is also applicable to networks of higher dimensions. The two-dimensional networks have almost identical stability boundaries for the fixed point and the bulk oscillation solutions as those in the one-dimensional networks (Fig 2B and S1 Fig). In the region above the period-doubling bifurcation curve of the bulk oscillation solution (Fig 2B, region with λ < −1), the two-dimensional networks have solutions similar to those in the one-dimensional networks, such as traveling waves (Fig 3A) and alternating bumps solutions (Fig 3B). In addition, we find other spatiotemporal patterns in the two-dimensional networks, such as spatially periodic stripe patterns that alternate in phase over time (Fig 3C).

**Fig 4:**
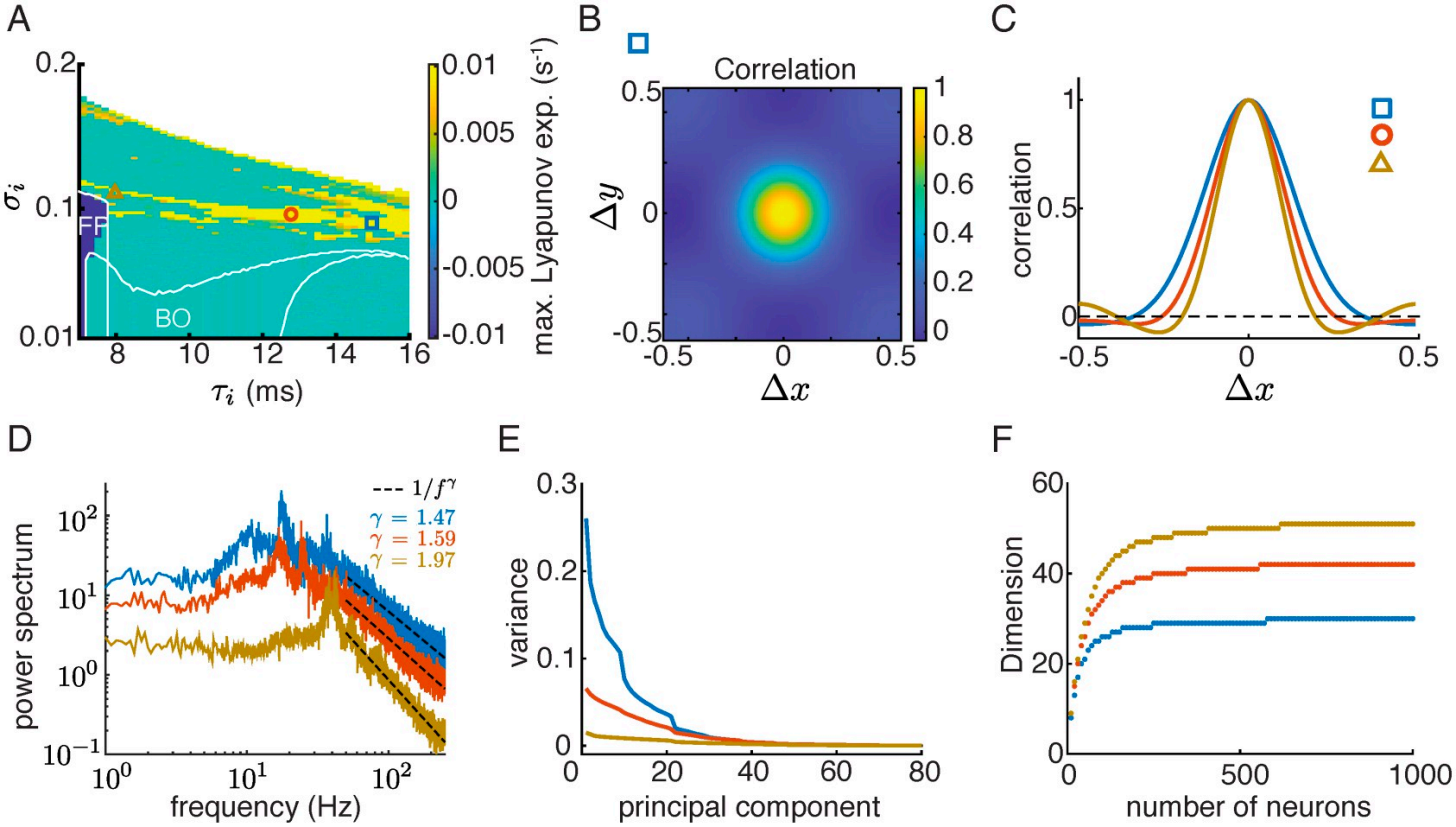
Population statistics of the chaotic solutions. **A**. The maximal Lyapunov exponent (MLE) as a function of the projection width (*σ*_*i*_) and the time constant (*τ*_*i*_) of the inhibitory population for the two-dimensional networks. The white curves are the stability borders of the parameter regions of stable fixed point (FP) and bulk oscillation (BO) solutions (same regions as in Fig 2B and S1 Fig). The color axis is limited at ±0.01 to better visualize the region of positive MLE’s (i.e. chaos region). **B**. The correlation between neuron activity as a function of distance in *x* and *y* directions (Eq 11) in a network labeled as a blue square in panel A. **C**. The correlation function along the *x* direction for three networks in the chaotic regime (Δ*y* = 0). The network parameters are denoted in panel A with corresponding symbols. **D**. Population averaged power spectrum of single-neuron rate activity represented in log-log scale (Eq 12). Dashed lines represent 1/*f*^*γ*^ scaling. **E**. The variance of the dominant principal components of population rate activity of 1000 randomly sampled neurons from the networks, averaged over 100 samples. **F**. The dimension of population activity saturates as the number of sampled neurons increases. The dimension is defined as the number of dominant principal components that account for 95% of the total variance. Snapshots of network activity from the three networks in C-F are shown in S3 Fig.

In particular, we find network solutions that are irregular in both space and time (Figs 3D, 1B and 1C). In these networks, neurons exhibit random like activity with large variability even though the network is deterministic. We verify that such irregular solutions are chaotic, meaning that a small perturbation leads to rapid divergence in network activity, by computing the maximal Lyapunov exponent (MLE) numerically (see Methods; [39]). The MLE measures the rate of separation between a perturbed trajectory and the original trajectory. The distance between the perturbed and and original trajectories grows as $e^{-\lambda_{\text{MLE}}t}\left|dz_{0}\right|$ where |*dz*_0_| is the size of the initial perturbation. A positive MLE means that the solution is chaotic, a negative MLE indicates a stable fixed point, and MLE = 0 indicates that the solution is periodic or quasi-periodic. We compute the MLE of network solutions over the parameter space of *σ*_*i*_ and *τ*_*i*_. We find chaotic solutions in a parameter region where the spatial scales of the excitatory and inhibitory projections are similar (*σ*_*i*_ ≈ *σ*_*e*_ = 0.1) and the time constant of the inhibitory neurons is large (*τ*_*i*_ > *τ*_*e*_ = 5 ms) (Fig 4A, yellow). Interestingly, anatomical measurements from sensory cortices find that excitatory and inhibitory neurons project on similar spatial scales [29, 30, 40]. In addition, the decay kinetics of inhibitory synaptic currents are also slower than the excitatory synaptic currents in physiology [28, 41, 42]. These results suggest that the network parameter region of chaotic dynamics is consistent with the anatomy and physiology of the cortex. In contrast, networks with one-dimensional spatial coupling have a restricted parameter region of chaos, suggesting that the two-dimensional spatial structure is important for generating chaos (S2 Fig).

### Population statistics of the chaotic solutions

Networks with chaotic dynamics intrinsically generate large variability in population activity. We next measure statistics of the population activity of networks in the chaotic regime and compare with cortical recordings. First, we compute the spatial correlations of population activity along *x* and *y* directions (Fig 4B and 4C, see Methods). The spatial correlations of the chaotic dynamics are roughly isotropic, and decrease with the distance between neurons (Fig 4B). A vanishing correlation at large distance and a positive Lyapunov exponent are the defining signatures of spatiotemporal chaos [43]. The decrease of correlation with cortical distance has been widely reported in population recordings across the cortex [19, 44–48]. Further, we found that the spatial decay rate of correlation depends on network parameters. Networks with larger *τ*_*i*_ have a broader range of correlation which remains positive over long distance (Fig 4C).

Second, we measure the power spectrum of the temporal variability of each neuron unit. We find that neurons in chaotic networks have broadband frequency power with peaks at around 20–40 Hz (Fig 4D). Beyond about 50 Hz, the power decays with frequency following a power law scaling (1/*f*^*γ*^) with exponent (*γ*) between 1.5 and 2 (Fig 4D dashed). This means that the neurons’ rate activities are not periodic as those in regular solutions, but exhibit considerable variability over a broad frequency range. This type of broadband frequency power has also been found in many neural recordings [49–51]. A power-law relationships is often regarded as a sign for self-organized critical states in network dynamics [52, 53]. However, power-law scaling can also arise from stochastic dynamics without being at critical states [54, 55]. The chaos in our networks does not result from a critical transition, which would imply scale-free temporal and spatial correlations. Instead, the chaotic dynamics in our networks have a characteristic temporal frequency and spatial scale of correlations.

Lastly, we examine the dimensionality of the chaotic solutions. We perform principal component analysis of the rate activity of 1000 randomly sampled neurons from the networks. The variance of each principal component is the eigenvalue of the covariance matrix of population rate over time, sorted from large to small. We find that the variability of the chaotic population rate concentrates in the first few tens of principal components (Fig 4E). We define the dimension of the population activity as the number of principal components that explains 95% of the variance. The dimension of chaotic solutions increases with the number of sampled neurons and saturates below 50 (Fig 4F), which is much lower than the number of neurons in the network (*N* = 10^4^). The low dimensional structure is a defining feature of cortical neural variability that has been recently demonstrated in multiple population recordings [20–24]. In contrast, the chaotic rate dynamics in disordered random networks have dimensions that increase linearly with network size [56].

### Transition to chaos through quasi-periodicity and intermittency

**Fig 5:**
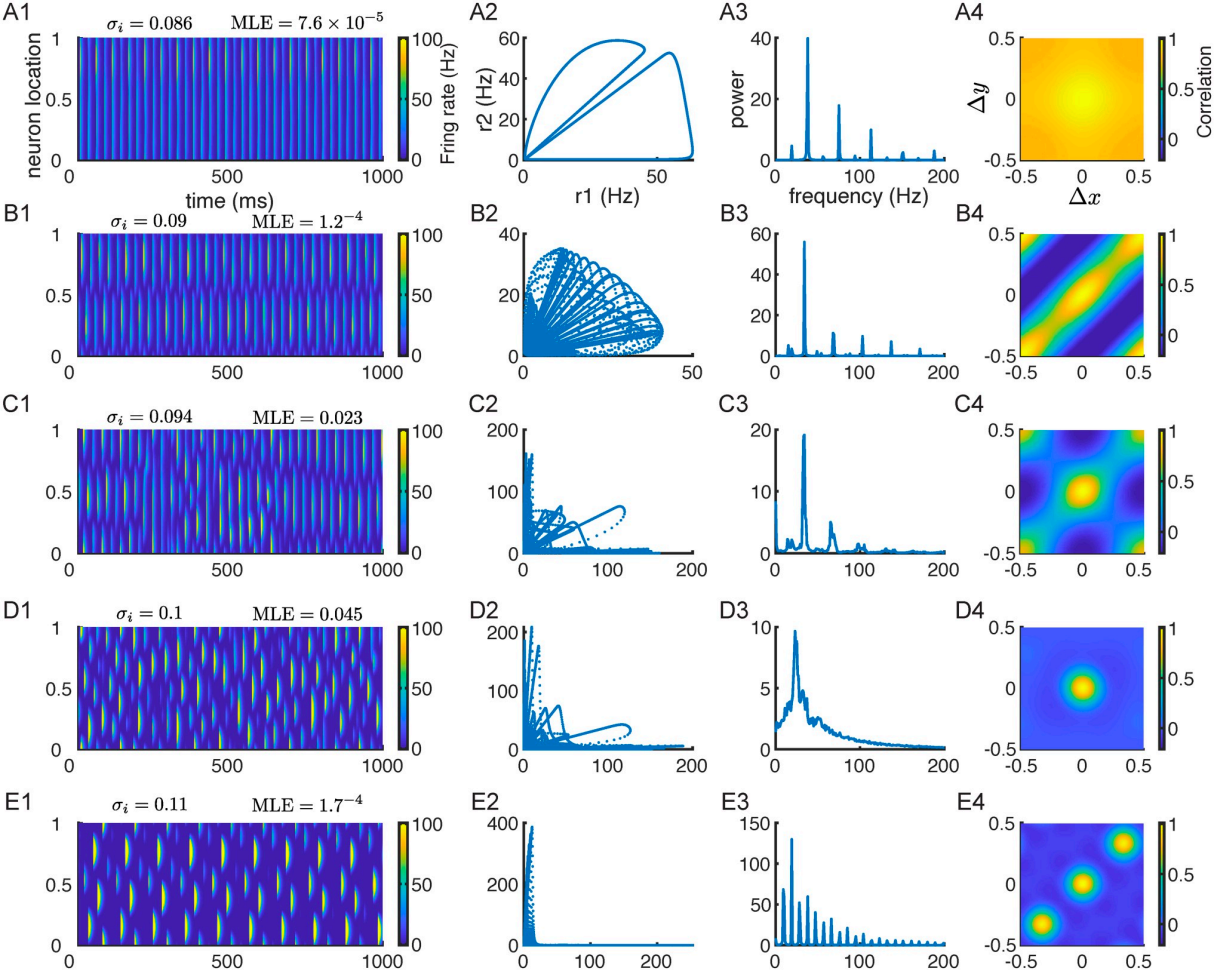
Transition to chaos as the inhibitory projection width (*σ*_*i*_) increases. **A1-E1**. Firing rate dynamics of excitatory neurons from a vertical slice of the network. The MLE of the solution is shown on top. **A2-E2**. Phase plots of firing rates of two excitatory neurons from different locations. **A3-E3**. Population averaged power spectrum of single-neuron rate activity (Eq 12). **A4-E4**. The correlation between neuron activity as a function of distance in *x* and *y* directions (Eq 11). The inhibitory time constant is fixed as *τ*_*i*_ = 11 ms.

To further investigate how network dynamics transition from a regular solution to chaos, we vary the inhibitory projection width (*σ*_*i*_) with fixed inhibitory time constant *τ*_*i*_. We find that the network transitions into chaos through quasi-periodicity and intermittency as *σ*_*i*_ increases (Fig 5). For each *σ*_*i*_, we show an example trial of rate dynamics from a vertical slice of the excitatory population (Fig 5 column 1), a phase plot of two excitatory neurons from different locations (Fig 5 column 2), the temporal power spectrum of single-neuron rate activity (Fig 5 column 3) and spatial correlations (Fig 5 column 4).

Beyond the period-doubling bifurcation of the bulk oscillation solution (Fig 2B and S1 Fig, magenta curves), the network has alternating bump solutions (Figs 5A and 3B). We can see that the solution is periodic both from the spatiotemporal activity from one slice of the network (Fig 5A1) and the phase plot of firing rates of two excitatory neurons (Fig 5A2). For this solution, the temporal power spectrum has sharp peaks at harmonic frequencies and the spatial correlation is large across all distances. As *σ*_*i*_ increases, the alternating bump solution loses stability and leads to quasi-periodic solutions with alternating stripe patterns (Fig 5B, similar to the solution in Fig 3C). The temporal power spectrum shows sharp frequency peaks (Fig 5B3) and the spatial correlation shows a diagonal stripe pattern (Fig 5B4). As *σ*_*i*_ further increases, the network shows intermittent behavior with alternating stripes interspersed with irregular activity (Fig 5C1, irregular activity around 500 ms). The MLE is positive for this network, indicating a chaotic solution. The spatial correlation peaks at the center and the opposite corners because the alternating stripes switch orientations from time to time (Fig 5C4). After a narrow parameter range of intermittent activity, the chaotic solution becomes more irregular with Gaussian-like spatial correlations that decay to zero at large distance (Fig 5D4). The temporal power spectrum contains a broad range of frequency power (Fig 5D3). Lastly, for larger *σ*_*i*_, chaotic solutions disappear and the network shows complex quasi-periodic activity (Fig 5E).

### Correlated input noise expands the chaotic regime

**Fig 6:**
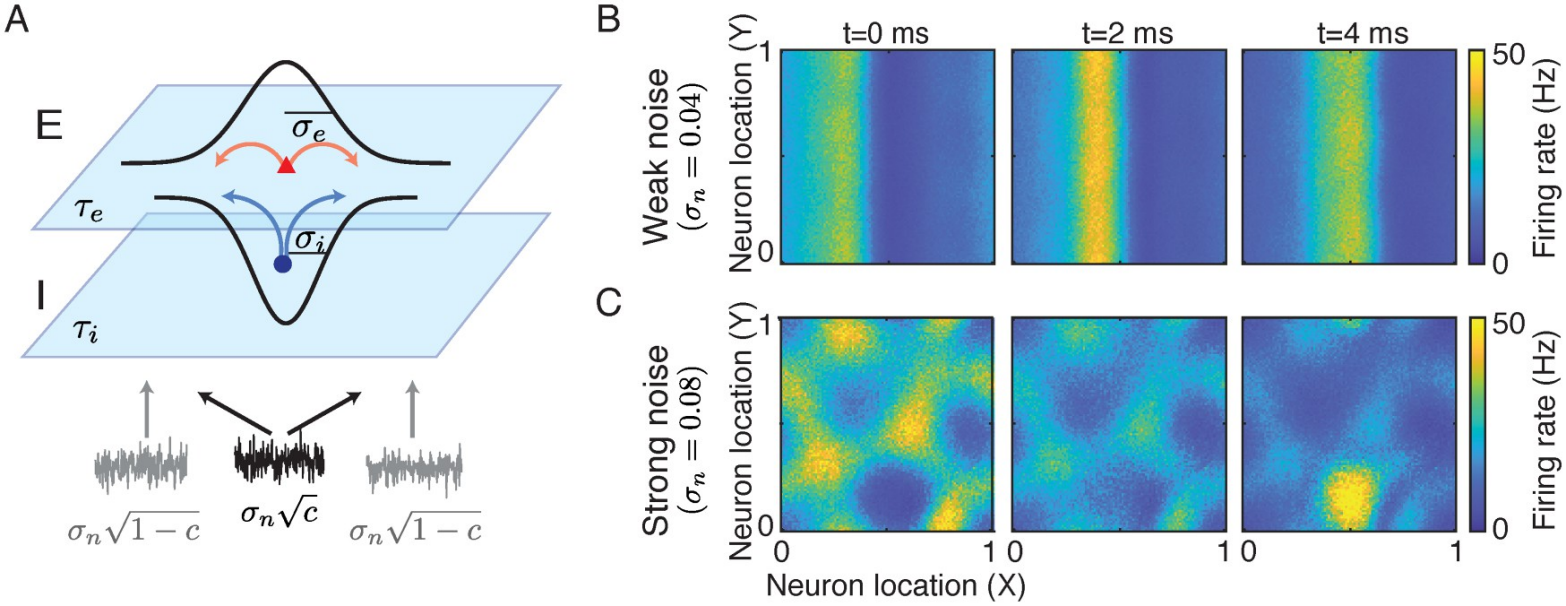
Two-dimensional networks with input noise. **A**. The input noise to each neuron consists of a correlated noise component (black) that is common for all neurons from the same population, and an independent noise component that is private to each neuron (gray). **B-C**. Snapshots of the firing rates of the excitatory population at three time frames for a network that generates traveling waves when there is no input noise (*τ*_*i*_ = 8, *σ*_*i*_ = 0.1, same parameters as in Fig 3A). The correlation of the input noise is *c* = 0.5, and the amplitude is *σ*_*n*_ = 0.04 (**B**) and *σ*_*n*_ = 0.08 (**C**).

We have, thus far, analyzed the behavior of fully deterministic networks. We now consider how input noise changes network dynamics. The input to neuron *k* from population *α* is

$$\begin{array}{c} I_{k}^{\alpha }=\mu_{\alpha }+\sigma_{n}(\sqrt{1-c}\cdot \eta_{k}^{\alpha }+\sqrt{c}\cdot \eta_{c}^{\alpha }), \end{array}$$ (5)

where *σ*_*n*_ is the standard deviation and *c* ∈ [0, 1] is the correlation coefficient of the input noise. The input noise to each neuron consists of two components: 1) an independent noise component, $\eta_{k}^{\alpha }$, which is private to each neuron *k*, and 2) a correlated noise component, $\eta_{c}^{\alpha }$, which is common for all neurons in population *α* ∈ {*e*, *i*} (Fig 6A; [57, 58]). Both noise components are modeled as Ornstein–Uhlenbeck processes with time constant *τ*_*n*_ = 5 ms (see Methods, Eq 13). The amplitude of the independent noise component is $\sigma_{n}\sqrt{1-c}$ and the amplitude of the correlated component is $\sigma_{n}\sqrt{c}$.

When the input noise is weak (small *σ*_*n*_), the regular solutions can roughly maintain their spatiotemporal patterns (Fig 6B). However, when the input noise is strong (large *σ*_*n*_), the patterns might be distorted or completely destroyed. For example, a network that produces traveling waves without input noise (Fig 3A) can still generate a noisy traveling wave pattern with weak noise (Fig 6B), but exhibits irregular patterns when input noise is strong (Fig 6C).

**Fig 7:**
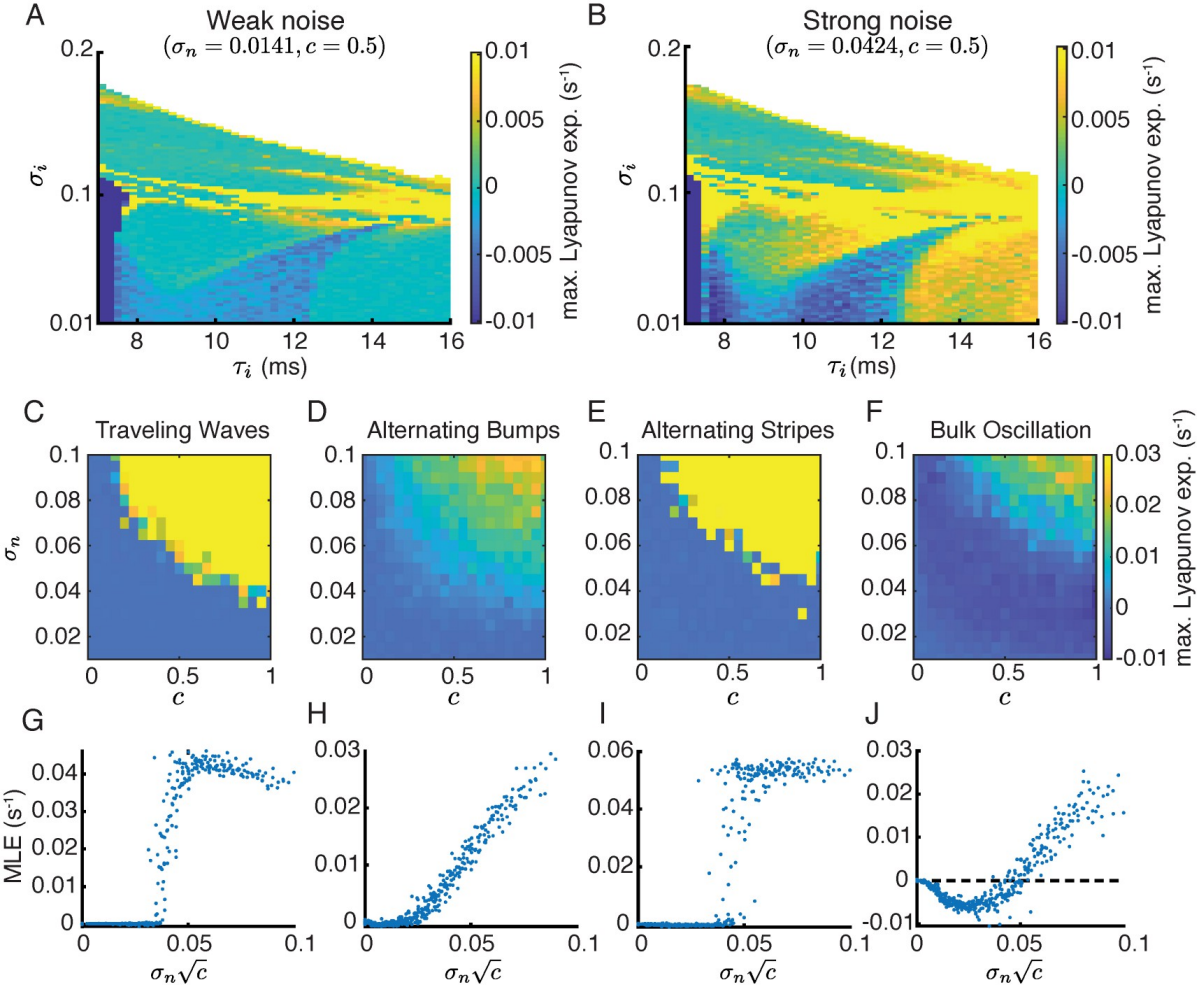
The maximal Lyapunov exponent (MLE) for the two-dimensional networks with input noise. **A-B**. The MLE map with weak (**A**, *σ*_*n*_ = 0.0141) and strong (**B**, *σ*_*n*_ = 0.0424) noise. The correlation of the input noise is *c* = 0.5. **C-F**. MLE as a function of the amplitude (*σ*_*n*_) and the correlation (*c*) of input noise, for four example networks that generate traveling waves (C; *τ*_*i*_ = 8, *σ*_*i*_ = 0.1, same as Fig 3A), or alternating bumps (D; *τ*_*i*_ = 9, *σ*_*i*_ = 0.06, same as Fig 3B), or alternating stripes (E; *τ*_*i*_ = 9, *σ*_*i*_ = 0.1, same as Fig 3C) or bulk oscillation (F; *τ*_*i*_ = 8, *σ*_*i*_ = 0.03) without noise. **G-J**. Same as panels C-F with MLE as a function of the amplitude of the correlated noise component, $\sigma_{n}\sqrt{c}$.

The irregular spatiotemporal patterns in networks with strong noise are similar to the chaotic solutions in deterministic networks (Fig 3A). We next compute the MLE of networks with frozen input noise in the parameter space of *σ*_*i*_ and *τ*_*i*_ (see Methods). We find that input noise can induce chaos in the spatially distributed networks. With correlated input noise, the parameter region of chaotic solutions expands as the amplitude of noise increases (compare yellow regions in Figs 4A and 7A and 7B). This suggests that a network receiving correlated input, for example from an upstream area, is more likely to generate chaotic dynamics than a network receiving static input without noise, which can potentially explain the prevalence of correlated activity across cortex.

In addition to inducing chaos, we find that noise can also synchronize bulk oscillations, reflected as negative MLE’s in the previously identified regime of bulk oscillations (light blue area in Fig 7A and 7B). A negative MLE of a noise driven system means that networks starting at different initial conditions would converge to the same network dynamical trajectory that depends only on the noise realization [59, 60].

Further, we investigate the impacts of the amplitude (*σ*_*n*_) and the correlation (*c*) of input noise on network dynamics. We compute the MLE of four examples of periodic solutions with varying *σ*_*n*_ and *c* (Fig 7C–7F). When there is no input noise (*σ*_*n*_ = 0), the MLE’s of these networks are zero, since they produce temporally periodic patterns. When driven by independent noise (*c* = 0), the MLE’s remain close to zero, which suggests that the periodic solutions are insensitive to independent noise. As *σ*_*n*_ and *c* increase, the MLE’s generally increase and become positive, indicating a transition to chaos (Fig 7C–7E). The bulk oscillation solution becomes synchronized with negative MLE for intermediate values of *σ*_*n*_ and *c* before transitioning to chaos (Fig 7F).

We next measure how MLE depends on the amplitude of the correlated noise component, $\sigma_{n}\sqrt{c}$ (Fig 6A). When plotting as a function of $\sigma_{n}\sqrt{c}$, the MLE’s for varying *σ*_*n*_ and *c* collapse to a single function curve (Fig 7G–7J). This suggests that the MLE of the noise driven system mainly depends on the amplitude of the correlated noise component. The transition to chaos can be sharp, as in the cases of traveling waves and alternating stripes (Fig 7G and 7I), or gradual, as in the cases of alternating bumps and bulk oscillation (Fig 7D and 7F). Therefore, we identify that it is the correlated noise component that is responsible for inducing chaos and synchronizing bulk oscillations in the two-dimensional networks. In contrast, the network dynamical patterns are insensitive to independent noise.

## Discussion

Variability in neural responses is prevalent in cortex. The structure of the variability shared among a neuron population has important consequences on the information processing of the network [61]. However, the circuit mechanism underlying neural variability remains unclear. In this work, we discover a new dynamical regime in spatially distributed neuronal networks where spatiotemporal chaos produce large magnitude of shared variability in population activity. The statistical properties of the spatiotemporal chaos are consistent with population recordings from cortex, such as the broadband frequency power in single neuron responses, distance-dependent correlations and the low dimensionality of population responses.

Our model incorporates a few generic biological features of cortical circuits. First, the synaptic connections between neurons are spatially organized, meaning that the connection probability between a pair of neurons strongly depends on the physical distance between them [26, 28–30, 40, 62]. Importantly, it has been found that the excitatory and inhibitory projections have a similar spatial footprints [29, 30, 40], which is also the network condition where we find chaotic dynamics. Second, the decay kinetics of inhibitory synaptic currents are much slower than the excitatory synaptic currents in physiology [28, 41, 42]. We find that slow inhibition is important to generate complex spatiotemporal dynamics. Lastly, we use a power-law transfer function of rate response, which has been found to well describe neuron responses measured in cat visual cortex [63]. Power-law transfer functions have also shown to be critical to explaining various features of neural responses from the visual cortex, such as contrast invariance of tuning, sublinear response summation and surround suppression [64–66]. We show that neural networks with these biological features generate complex spatiotemporal dynamics whose population statistics match those from cortical recordings during the awake state.

Rate chaos in neuronal networks has been widely studied using random recurrent networks, where the connection weights from each neuron follow a Gaussian distribution with zero mean [9]. The network solution transitions from a stable fixed point to chaos when the variance of connection weights exceeds a critical value. Similar transition to chaos is also observed in networks with separate excitatory and inhibitory populations [67] and in spiking neuron networks [68]. In contrast, in spatially distributed networks, chaos appears after several solutions of different spatiotemporal patterns lose stability. As *σ*_*i*_ increases, the bulk oscillation solutions transition to alternating bump solutions, which transition to alternating stripes or traveling waves, and then to chaos (Fig 5). Further theoretical analysis is needed to elucidate the transition to chaos in spatial networks.

The spatiotemporal chaos in our networks has several distinct features from the chaotic solutions in random neuronal networks [9, 11, 69]. First, in networks of unstructured random connectivity, the correlations among neurons vanish as network size becomes large (scales as $1/\sqrt{N}$ with *N* being the network size) [11, 70]. Hence, those networks do not generate correlated population patterns, while the chaos in spatial networks produce distance-dependent correlations. Second, the dimensionality of population activity in random networks has been found to be high and increases linearly with network size [56]. This is in contrast with the low dimensional structure of the spatiotemporal chaos found in spatial networks (Fig 4F) and population activity found in experimental data [20–24]. Lastly, previous work showed that time varying inputs, such as independent white noise and oscillatory inputs, suppress chaos [11, 56, 69, 71, 72], while we found that the chaotic solutions in spatial networks are insensitive to independent noise and that chaos can be induced by correlated noise (Fig 7). This provides a testable prediction that a spatially global and time-varying stimulation can desynchronize strong oscillations and increase irregularity in population activity.

Spatiotemporal chaos in distributed excitable systems has been studied in different models, such as fluid turbulence, coupled oscillators and coupled maps [43, 73]. Most of the models are reaction-diffusion models and chaos is induced by diffusion. In contrast, our model is a system of integro-differential equations with non-local coupling. In neural network models, spatiotemporal chaos has been demonstrated in networks with local coupling [74, 75]. In [74] spatiotemporal chaos occurs in networks with a large diffusion coefficient of the inhibitory membrane potential, which models for local electrical coupling such as gap junctions. In contrast, we find chaos when the inhibitory neurons have a similar spatial scale as the excitatory neurons. The spatiotemporal chaos in their networks is caused by an interplay between Turing and Hopf instabilities of the fixed point solution, where both a nonzero and the zero wave number lose stability. This is not the case in our model, where both the fixed point and the bulk oscillation solutions are destabilized at multiple wave numbers in the parameter region of chaos (S4B and S4C Fig). Similar to our model, they also need the time constant of the inhibitory population to be large. [75] studies networks with local coupling (such as nearest neighbor coupling) among excitatory neurons and distance-dependent delays. They show that spatiotemporal chaos can appear when the network size is large. Similar to our model, they also find spatiotemporal chaos after the bulk oscillation solution loses stability and find intermittent solutions before the appearance of spatiotemporal chaos. Delays have been shown to generate various spatiotemporal patterns [76] and may play a similar role to the inhibitory time constant (*τ*_*i*_) in our model. In neither of these works are the effects of noisy inputs studied.

Several recent models have studied chaos in random neuronal networks with structured connectivity. For example, networks with a low rank connectivity component in addition to a random component can generate low dimensional coherent chaos, which can be utilized for complex computations [77, 78]. Networks with cell-type-dependent distributions of connections can produce chaos with multiple modes of autocorrelation functions of individual neurons [79]. In this work, we demonstrate that networks with two-dimensional spatial couplings and no random connectivity can also generate chaos which resides in a low-dimensional state space. How random connectivity in combination with spatially ordered connectivity affect chaos remain to be studied in future work.

Chaotic dynamics in neuronal networks offer a rich “reservoir” of population activity patterns, which can be utilized to learn a target output function or accomplish complex neural computations [12, 33, 80–82]. Near the transition of chaos, networks can generate slow dynamics which are important for temporal integration and necessary for many behavioral tasks [13, 83]. Information diffusion within a network has been found to be high in the regime of spatiotemporal chaos suggesting rapid mixing of information [75]. Here we find a new type of spatiotemporal chaos in networks where the connectivity features are consistent with cortical anatomy. It would be fruitful to explore the computational benefits of such chaos in spatially distributed networks.

## Methods

### Stability of fixed point solutions

Linearization around the fixed point solution $\left({\bar{r}}_{e},{\bar{r}}_{i}\right)$ of Eqs 1 and 2 in Fourier space gives a Jacobian matrix at each spatial Fourier mode:

$$\begin{array}{c} J\left(\vec{n}\right)=(\begin{array}{cc} \left(-1+L_{e}W_{ee}\tilde{g}\left(\vec{n},\sigma_{e}\right)\right)/\tau_{e} & L_{e}W_{ei}\tilde{g}\left(\vec{n},\sigma_{i}\right)/\tau_{e}\\ L_{i}W_{ie}\tilde{g}\left(\vec{n},\sigma_{e}\right)/\tau_{i} & \left(-1+L_{i}W_{ii}\tilde{g}\left(\vec{n},\sigma_{i}\right)\right)/\tau_{i} \end{array}), \end{array}$$ (6)

where $\vec{n}$ is the Fourier mode, $\tilde{g}\left(\vec{n},\sigma_{\alpha }\right)=\text{exp}(-2||\vec{n}{\left|\right|}^{2}\pi^{2}\sigma_{\alpha }^{2})$ is the Fourier Gaussian kernel, and $L_{\alpha }=\phi^{\prime }\left(u_{\alpha }^{*}\right)$ evaluated at the fixed point $u_{\alpha }^{*}=W_{\alpha e}{\bar{r}}_{e}+w_{\alpha i}{\bar{r}}_{i}+\mu_{\alpha }$, *α* = {*e*, *i*}.

The fixed point is stable if all eigenvalues of $J(\vec{n})$ have negative real part for any $\vec{n}$ (Fig 2B). Note that the stability only depends on the wave number $k=\left|\right|\vec{n}\left|\right|$.

### Stability of bulk oscillation solutions

To analyze the stability of bulk oscillation solutions, we linearize around the time dependent limit cycle solution, $\left({\bar{r}}_{e}\left(t\right),{\bar{r}}_{i}\left(t\right)\right)$, and obtain the Jacobian matrix at each Fourier mode, which is also periodic in time [37, 38]:

$$\begin{array}{c} J\left(\vec{n};t\right)=(\begin{array}{cc} \left(-1+L_{e}\left(t\right)W_{ee}\tilde{g}\left(\vec{n},\sigma_{e}\right)\right)/\tau_{e} & L_{e}\left(t\right)W_{ei}\tilde{g}\left(\vec{n},\sigma_{i}\right)/\tau_{e}\\ L_{i}\left(t\right)W_{ie}\tilde{g}\left(\vec{n},\sigma_{e}\right)/\tau_{i} & \left(-1+L_{i}\left(t\right)W_{ii}\tilde{g}\left(\vec{n},\sigma_{i}\right)\right)/\tau_{i} \end{array}), \end{array}$$ (7)

where $L_{\alpha }\left(t\right)=\phi^{\prime }\left(u_{\alpha }^{*}\left(t\right)\right)$ evaluated along the limit cycle solution $u_{\alpha }^{*}=W_{\alpha e}{\bar{r}}_{e}\left(t\right)+w_{\alpha i}{\bar{r}}_{i}\left(t\right)+\mu_{\alpha }$, *α* = {*e*, *i*}. The perturbation of rate, $\delta \tilde{\text{r}}\left(\vec{n}\right)$, at Fourier mode, $\vec{n}$, follows a linear system with periodic coefficients:

$$\begin{array}{c} \frac{d\delta \tilde{\text{r}}\left(\vec{n}\right)}{dt}=J\left(\vec{n};t\right)\cdot \delta \tilde{\text{r}}\left(\vec{n}\right). \end{array}$$ (8)

We obtain a principal fundamental matrix solution of Eq 8 by solving $X^{\prime }=J\left(\vec{n};t\right)X$ with initial conditions *X*(0) = *I*, where *I* is the identity matrix. The stability of the bulk oscillation solution is then determined by the eigenvalues of the monodromy matrix,

$$\begin{array}{c} M\left(\vec{n}\right)=X\left(\vec{n};T\right), \end{array}$$ (9)

where *T* is the period of the bulk oscillation solution. If any of the eigenvalues of $M(\vec{n})$ have magnitude greater than 1 at some Fourier model $\vec{n}$, then the bulk oscillation will lose stability with spatial mode $\vec{n}$. A real eigenvalue less than -1 indicates a period-doubling bifurcation, while a real eigenvalue larger than 1 suggests a pattern formation with the same period as the bulk oscillation [37].

### Maximal Lyapunov exponent

The maximal Lyapunov exponent (MLE) is computed numerically using the method by [39]. We first simulate the network long enough such that the solution has converged to an attractor. We denote the solution trajectory as $\vec{R}\left(t\right)={\left(r_{e}\left(x,t\right),r_{i}\left(x,t\right)\right)}^{T}$.

We continue simulating the trajectory for *n* time points, {*t*_0_, *t*_1_, …, *t*_*n*_}, with step size Δ*t*. At the initial time point, *t*_0_, we perturb the trajectory, $\vec{R}\left(t\right)$, by $d{\vec{z}}_{0}$, which is in a random direction and has a small magnitude $|d{\vec{z}}_{0}|=dM$. We integrate the same model system with the perturbed initial condition, $\vec{R}\left(t_{0}\right)+d{\vec{z}}_{0}$, by one time step, Δ*t*, and obtain the perturbed trajectory, ${\vec{R}}_{p}\left(t_{1}\right)$. The separation between the two trajectories is $D{\vec{z}}_{1}={\vec{R}}_{p}\left(t_{1}\right)-\vec{R}\left(t_{1}\right)$. Then we choose the perturbation at the next time step as $d{\vec{z}}_{1}=\frac{D{\vec{z}}_{1}}{|D{\vec{z}}_{1}|}dM$. In this way, the perturbation in each time point has a magnitude of *dM*, and is in the same direction as the separation between the original and the perturbed trajectories at the previous time step. We then integrate the same model system with the perturbed initial condition, $\vec{R}\left(t_{1}\right)+d{\vec{z}}_{1}$, by one time step and obtain ${\vec{R}}_{p}\left(t_{2}\right)$. We repeat this procedural *n* steps and obtain a sequence of trajectory separations, $(D{\vec{z}}_{i}{)}_{i=0}^{n}$. Lastly, the Maximal Lyapunov exponent is computed as

$$\begin{array}{c} \text{MLE}=\frac{1}{n\Delta t}\sum_{i=1}^{n}\text{log}(|D{\vec{z}}_{i}\left|/dM\right), \end{array}$$ (10)

where *n* = 10^6^ and Δ*t* = 0.01*s*. The MLE converges at large *n* (S5 Fig).

### Spatial correlation

The spatial correlations in Fig 4B and 4C are defined as the Pearson correlation coefficient of *r*_*e*_, for each neural distance (Δ*x*, Δ*y*):

$$\begin{array}{c} C\left(\Delta x,\Delta y\right)=\frac{{\left\langle (r_{e}\left(x+\Delta x,y+\Delta y,t\right)-\langle r_{e}{\left(x+\Delta x,y+\Delta y,t\right)\rangle }_{t})(r_{e}\left(x,y,t\right)-\langle r_{e}{\left(x,y,t\right)\rangle }_{t})\right\rangle }_{x,y,t}}{\text{Var}(r_{e}\left(x,y,t\right))}\;, \end{array}$$ (11)

where 〈⋅〉_*t*_ is average over time and 〈⋅〉_*x*,*y*,*t*_ denotes average over time and space.

### Power spectrum

The power spectrum in Fig 4D is defined as:

$$\begin{array}{c} S={\left\langle |F{\left(r_{e}-\langle r_{e}\right\rangle }_{t}){|}^{2}\right\rangle }_{x,y}\;, \end{array}$$ (12)

where the function *F* is the Fourier transform in time.

### Input noise

The input noise terms $\eta_{k}^{\alpha }$ and $\eta_{c}^{\alpha }$ (*α* ∈ {*e*, *i*}) in Eq 5 are modeled as independent Ornstein–Uhlenbeck processes:

$$\begin{array}{c} \tau_{n}d\eta =-\eta dt+dW, \end{array}$$ (13)

where *W* is a Wiener process, and the noise time constant is *τ*_*n*_ = 5 ms.

### Network parameters

Unless specified otherwise, the network parameters are *W*_*ee*_ = 80, *W*_*ei*_ = −160, *W*_*ie*_ = 80, *W*_*ii*_ = −150, *τ*_*e*_ = 5 ms, *σ*_*e*_ = 0.1, *μ*_*e*_ = 0.48 and *μ*_*i*_ = 0.32. *τ*_*i*_ and *σ*_*i*_ vary in each figure and are specified in figure axes and captions. The number of neurons in the two dimensional network are *N* = 100 × 100 for each of the excitatory and inhibitory populations. In the one dimensional network *N* = 100 for each of the populations.

## Supporting information

S1 Fig Related to Fig 3. Phase diagram of networks with two dimensional spatial coupling. Same format as Fig 2B in the main text. The letters mark the locations of the parameters used in Fig 3A–3D.
(TIFF)

S2 Fig Related to Fig 4. The maximal Lyapunov exponents of networks with one dimensional spatial coupling. Same format as Fig 4A. The maximal Lyapunov exponent as a function of the projection width (*σ*_*i*_) and the time constant (*τ*_*i*_) of the inhibitory neurons. The white curves are the stability borders of the parameter regions of stable fixed point (FP) and bulk oscillation (BO) solutions (same regions as in Fig 2B).
(TIFF)

S3 Fig Related to Fig 4. Snapshots of the firing rates of the excitatory population at five time frames from the three chaotic solutions in Fig 4. There is no qualitative difference in the activity.
(TIFF)

S4 Fig Related to Fig 4. **A**. The maximal Lyapunov exponent (MLE) as a function of the projection width (*σ*_*i*_) and the time constant (*τ*_*i*_) of the inhibitory population for the two-dimensional networks. Same as Fig 4A in the main text. **B**. The number of unstable wave numbers from the stability analysis of the bulk oscillation solution. **C**, The number of unstable wave numbers from the stability analysis of the fixed point solution. Orange dots in panels B and C denote the chaos region shown in panel A with MLE>0.005. **A-C**. Same network parameter as in Fig 4 of the main text. **D-F**. Same parameters as A-C except that *W*_*ie*_ is changed from 80 to 100.
(TIFF)

S5 Fig Related to Methods: Maximal Lyapunov exponent. Convergence of the maximal Lyapunov exponent as a function of the number of perturbations *n* (Eq 10). Each curve is the MLE for one solution. There are 10 chaotic solutions (MLE>0.005) and 5 periodic solutions (MLE<0.002).
(TIFF)
