# Supplementary figures and images for: Chaotic dynamics in spatially distributed neuronal networks generate population-wide shared variability
Source: PLoS Comput Biol. 2023 Jan 10;19(1):e1010843. doi: 10.1371/journal.pcbi.1010843 (PMC9870129; doi:10.1371/journal.pcbi.1010843)

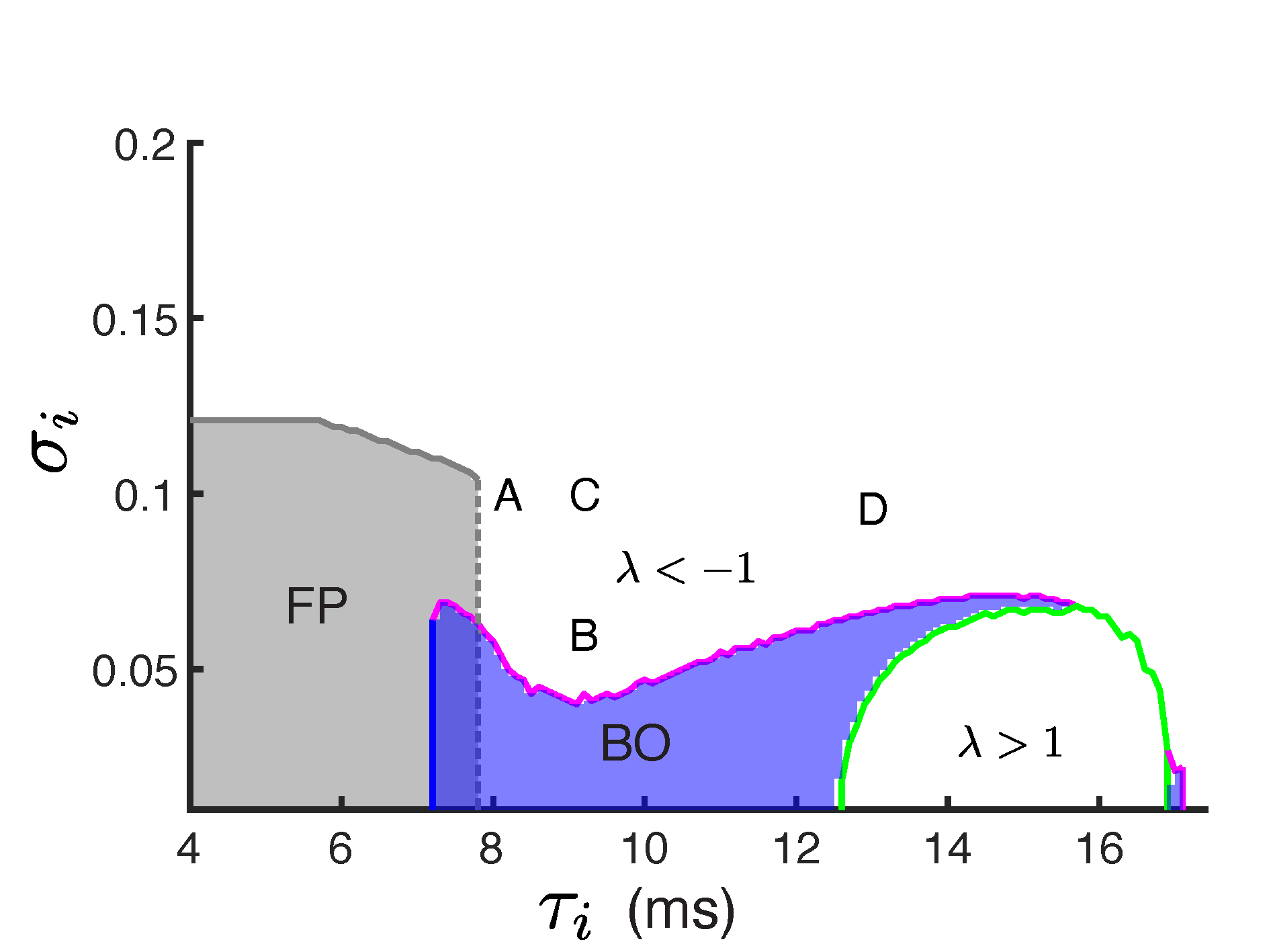

Supplement: S1 Fig — Phase diagram of networks with two dimensional spatial coupling. Same format as Fig 2B in the main text. The letters mark the locations of the parameters used in Fig 3A–3D. (TIFF) [file pcbi.1010843.s001.tiff]

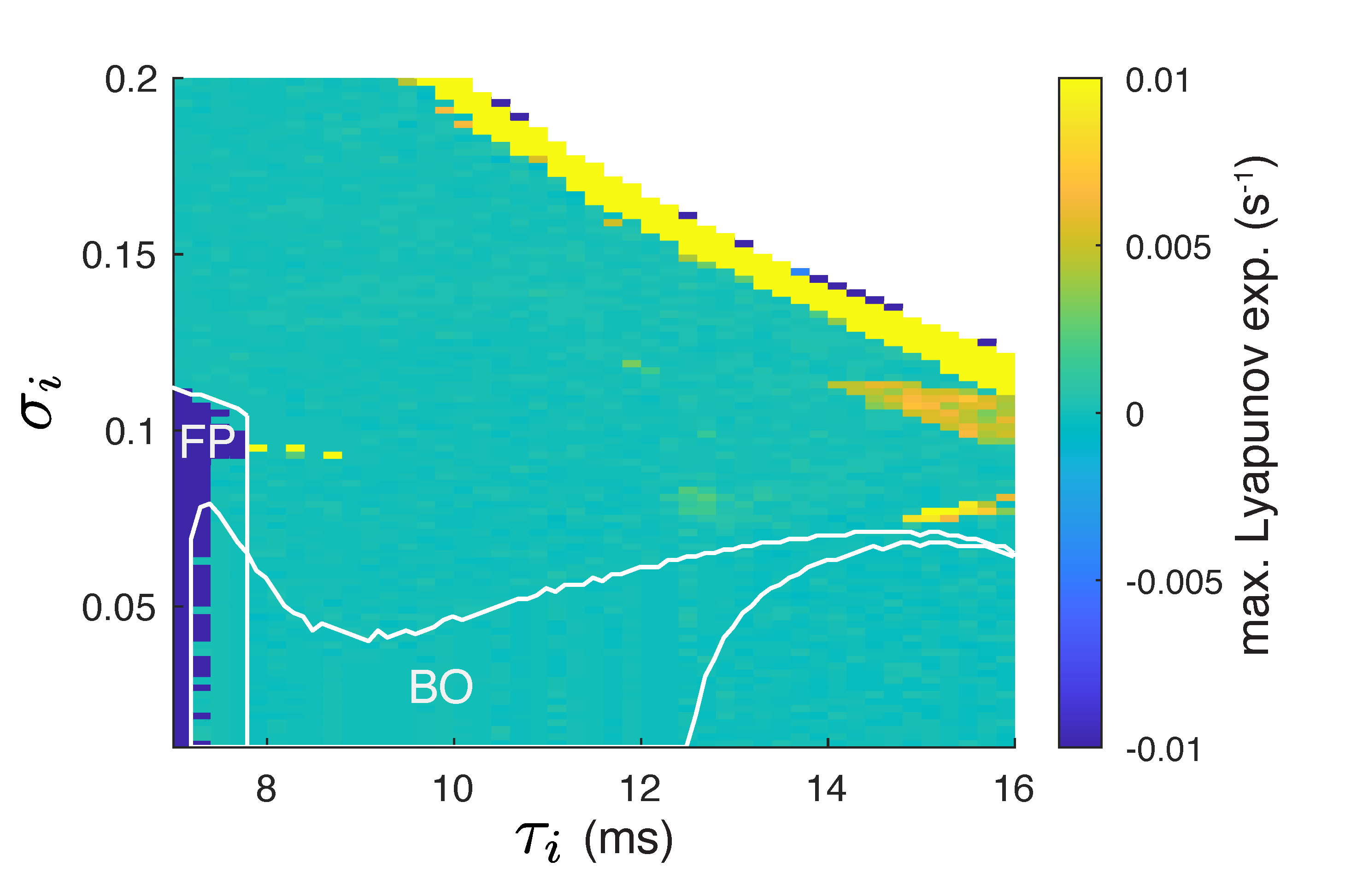

Supplement: S2 Fig — The maximal Lyapunov exponents of networks with one dimensional spatial coupling. Same format as Fig 4A. The maximal Lyapunov exponent as a function of the projection width (σi) and the time constant (τi) of the inhibitory neurons. The white curves are the stability borders of the parameter regions of stable fixed point (FP) and bulk oscillation (BO) solutions (same regions as in Fig 2B). (TIFF) [file pcbi.1010843.s002.tiff]

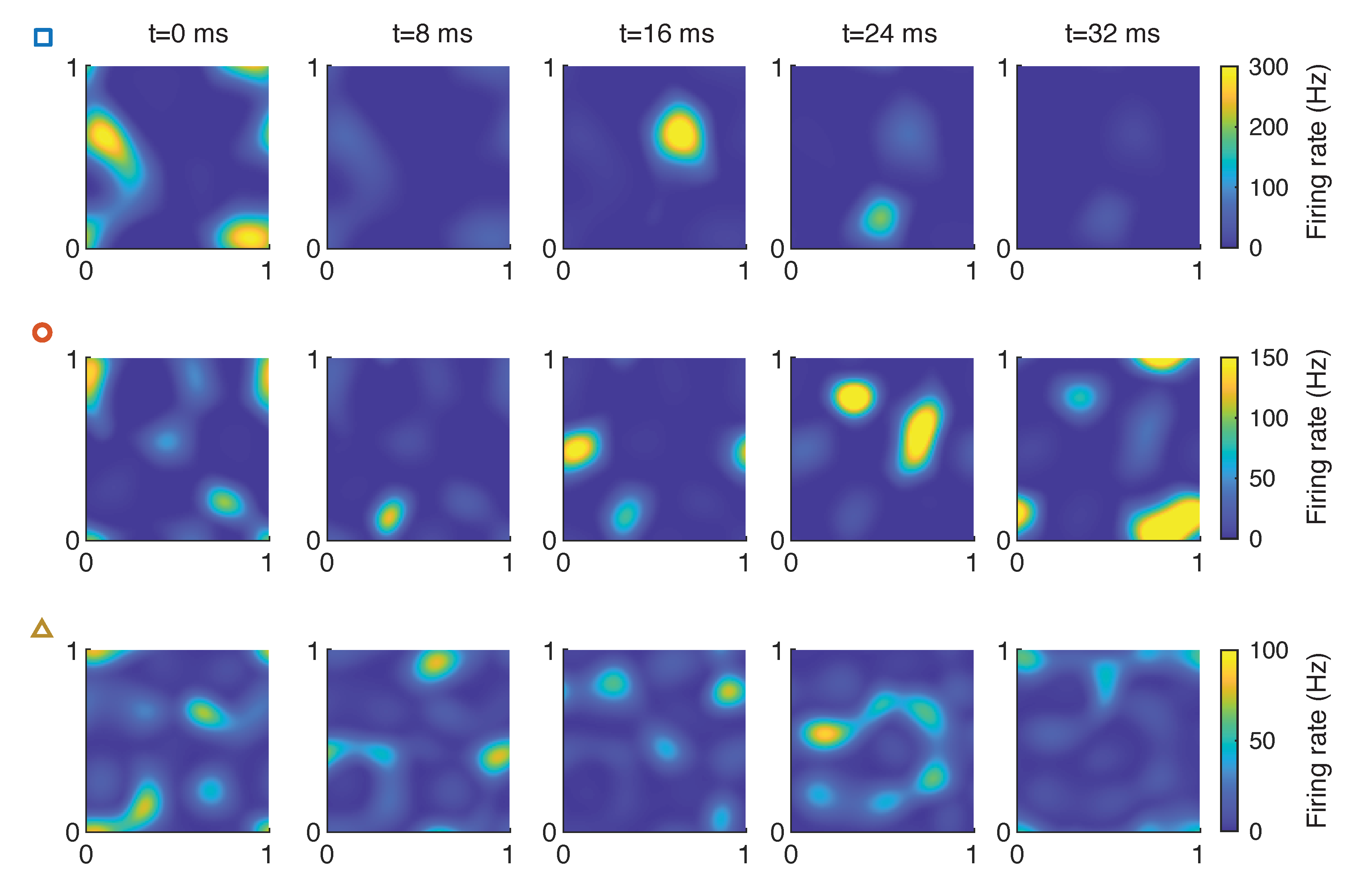

Supplement: S3 Fig — Snapshots of the firing rates of the excitatory population at five time frames from the three chaotic solutions in Fig 4. There is no qualitative difference in the activity. (TIFF) [file pcbi.1010843.s003.tiff]

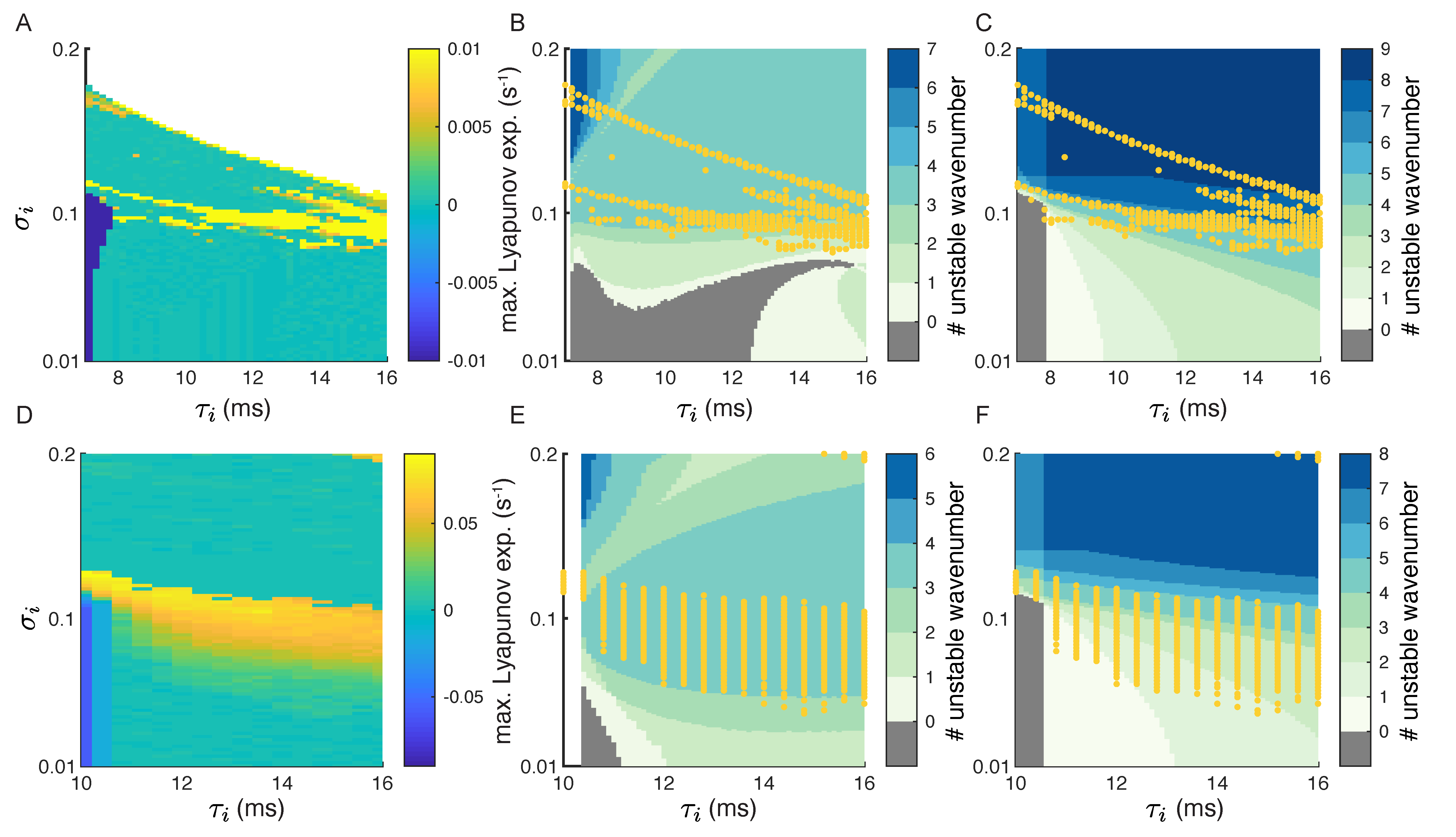

Supplement: S4 Fig — A. The maximal Lyapunov exponent (MLE) as a function of the projection width (σi) and the time constant (τi) of the inhibitory population for the two-dimensional networks. Same as Fig 4A in the main text. B. The number of unstable wave numbers from the stability analysis of the bulk oscillation solution. C, The number of unstable wave numbers from the stability analysis of the fixed point solution. Orange dots in panels B and C denote the chaos region shown in panel A with MLE>0.005. A-C. Same network parameter as in Fig 4 of the main text. D-F. Same parameters as A-C except that Wie is changed from 80 to 100. (TIFF) [file pcbi.1010843.s004.tiff]

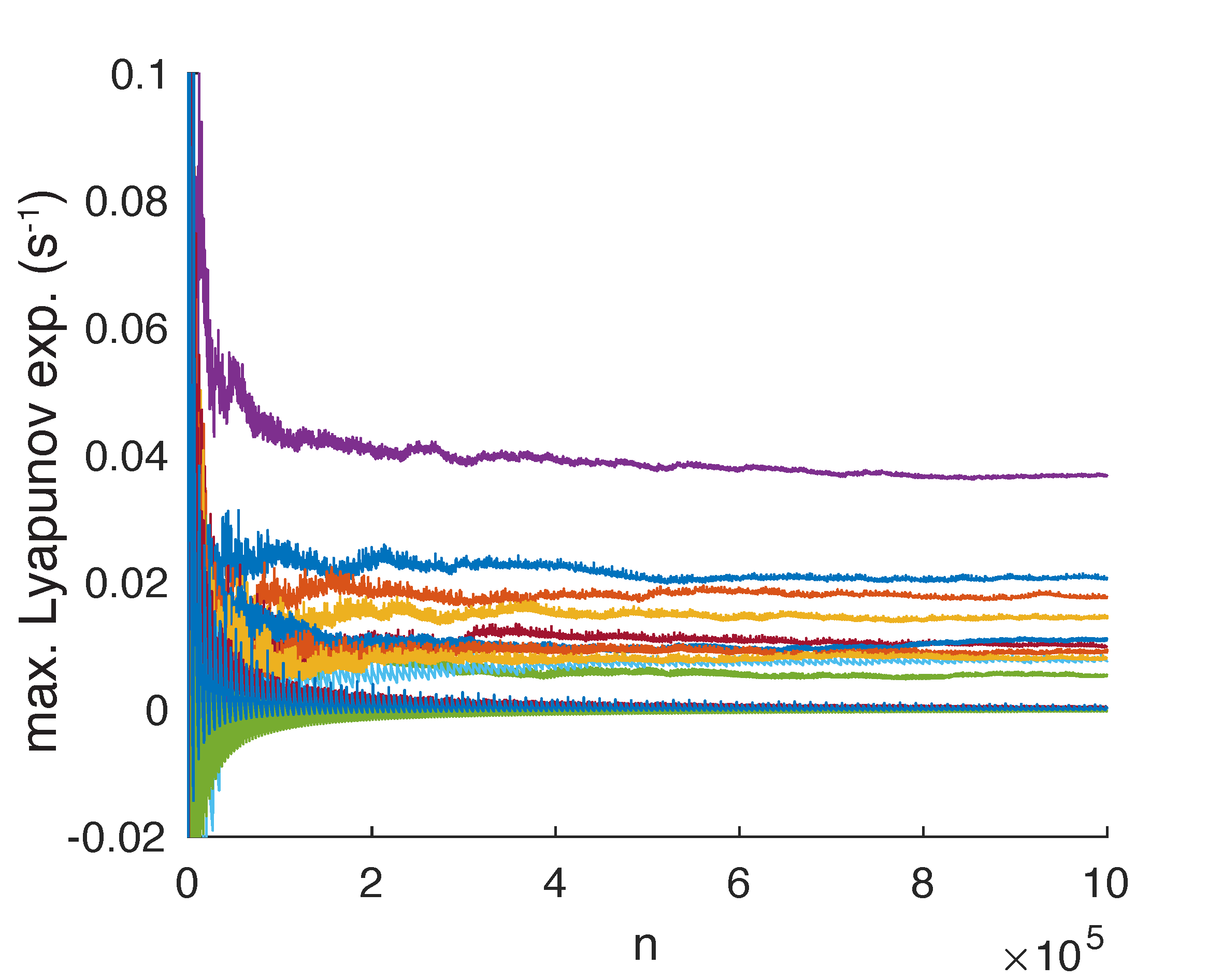

Supplement: S5 Fig — Convergence of the maximal Lyapunov exponent as a function of the number of perturbations n (Eq 10). Each curve is the MLE for one solution. There are 10 chaotic solutions (MLE>0.005) and 5 periodic solutions (MLE<0.002). (TIFF) [file pcbi.1010843.s005.tiff]
